# Nitric oxide-releasing balloon inflation fluid for Foley catheters to prevent catheter-associated urinary tract infections

**DOI:** 10.1128/spectrum.00818-26

**Published:** 2026-06-29

**Authors:** Ryan Kauffman, Katherine McCombs, Jianfeng Wu, Yao Lee, Ingrid L. Bergin, Spencer Amacher, Chuanwu Xi, Mark E. Meyerhoff, Gergely Lautner, Alvaro Rojas-Pena, Karthik Ramani, Orsolya Lautner-Csorba

**Affiliations:** 1 Department of Surgery, Robert H. Bartlett Extracorporeal Life Support (ECLS) Lab, University of Michigan 199668, Ann Arbor, Michigan, USA; 2 Department of Chemistry, University of Michigan 205700, Ann Arbor, Michigan, USA; 3 School of Public Health, University of Michigan 51329, Ann Arbor, Michigan, USA; 4 Unit for Laboratory Animal Medicine, Pathology Core, University of Michigan 1259, Ann Arbor, Michigan, USA; 5 Department of Pharmaceutical Sciences, University of Michigan 536139, Ann Arbor, Michigan, USA; 6 Department of Surgery, Section of Transplantation, University of Michigan 199668, Ann Arbor, Michigan, USA; 7 Division of Nephrology, University of Michigan 274061, Ann Arbor, Michigan, USA; Illinois State University, Normal, Illinois, USA

**Keywords:** nitric oxide, Foley catheter, urinary tract infection, inflation fluid

## Abstract

Catheter-associated urinary tract infections (CAUTIs) are the most prevalent nosocomial infections. These infections impose a significant financial burden on the healthcare system and are responsible for approximately 15,000 deaths each year in the US. CAUTIs are caused by free-floating bacteria that adhere to the surface of Foley catheters and the patient’s uroepithelium, forming protective biofilms that enhance bacterial survival and resistance. In this study, a nitric oxide (NO)-releasing liquid formulation is used to inflate the Foley catheter balloon, aiming to inhibit biofilm formation and reduce the risk of CAUTIs. The solution of S-nitrosoglutathione (GSNO), an endogenous NO donor molecule, was used as the catheter inflation fluid. To assess the NO-release profile, the GSNO was dissolved in various pH-adjusted (pH 6.5) aqueous media (deionized water, phosphate-buffered saline, and 2-[4-(2-hydroxyethyl)piperazine-1-yl]ethanesulfonic acid [HEPES] buffers). The GSNO/HEPES formulation sustained NO fluxes through the inflated balloon wall above 0.5 × 10⁻¹⁰ mol·min⁻¹·cm⁻² for up to 10 days, as well as significantly reduced the planktonic bacteria counts and surface colonization against *Escherichia coli*. The safety of the optimized GSNO formulation was evaluated *in vivo* in a 7-day preclinical porcine model. The formulation showed no signs of NO-related toxicity. Overall, this study presents a promising, translatable approach to substantially reducing the risk of CAUTIs in healthcare settings.

**IMPORTANCE:**

Catheter-associated urinary tract infections (CAUTIs) are the most frequent hospital-acquired infections and pose serious risks for patients with Foley catheters in hospitals, nursing homes, and other care facilities. These infections lead to significant mortality and healthcare expenses and are especially difficult to prevent due to bacterial biofilms on catheter surfaces. Our study introduces a novel solution, a nitric oxide (NO)-releasing balloon inflation fluid using S-nitrosoglutathione (GSNO), that continuously releases antimicrobial NO for up to 10 days. This approach significantly reduces bacterial counts and surface colonization by *Escherichia coli*, a main cause of CAUTIs, while demonstrating safety in preclinical animal testing. Our findings suggest that this strategy could reduce infection risk and improve outcomes for catheterized patients across diverse healthcare settings.

## INTRODUCTION

Urinary tract infections (UTIs) are the most common nosocomial infections, with indwelling urinary catheters being a major risk factor for their development (1, 2). Catheter-associated urinary tract infections (CAUTIs) account for approximately 0.5–0.7 million hospital-acquired infections annually, leading to various complications such as cystitis, pyelonephritis, gram-negative bacteremia, prostatitis, epididymitis, urosepsis, and septicemia and resulting in around 10,000 deaths each year (1–3). In the United States, 95% of UTIs in medical intensive care units are CAUTIs (4). When using a Foley catheter, bacteriuria develops at a daily incidence rate of approximately 5%–10% (5). Once established, CAUTIs are challenging to treat because of the formation of bacterial biofilms that grant resistance to systemic antibiotics (6–8). Despite the excretion of high concentrations of inherent antimicrobial agents in the urine, effectively clearing bacteriuria often requires catheter removal and systemic antibiotic therapy. Numerous strategies have been explored to reduce the incidence of CAUTIs, including the implementation of fully closed drainage systems and prophylactic use of systemic antibiotics (9–11). Current technologies and devices have been developed to prevent biofilm formation, such as coatings with silver, gendine, hydrogel, triclosan, antibiotics, quorum-sensing inhibitors, and biological coatings (bacteria or bacteriophages) (12). Although these technologies generally demonstrate a reduced risk of bacteriuria, drawbacks include higher costs, potential for promoting antibiotic resistance (in the case of antibiotic-coated catheters), and inconsistent clinical effectiveness. Furthermore, the financial burden associated with CAUTI is quite significant, resulting in an incremental cost of $6.9B–$10.0B to the US healthcare system annually (13).

Nitric oxide (NO) is an endogenously formed radical known to play a key role in the immune response and in preventing infection. Studies have shown that low nanomolar concentrations of NO efficiently disperse biofilm formed by a variety of bacterial strains (14–20). In addition, medical devices coated, impregnated, or inflated with NO donors have been shown to decrease medical device-associated infections (21–23). Previously, an entire Foley catheter was solvent-impregnated with a NO donor, S-nitroso-N-acetylpenicillamine (SNAP), to prevent CAUTI. The impregnated Foley catheter demonstrated relevant antimicrobial properties against *Staphylococcus epidermidis* and *Proteus mirabilis* (23). However, the catheter’s mechanical properties (e.g., flexibility) were not assessed, raising concerns about potential crystal formation within the catheter material caused by the NO donor, SNAP (24, 25). Previous studies tested NO-releasing inflation fluid utilizing acidified nitrite (21, 26) and DETA NONOate (21) solutions. These formulations demonstrated substantial antimicrobial effects against several bacterial strains. Interestingly, while Carlsson et al. (21) reported that the acidified nitrite solution was effective against *Escherichia coli*, Kishikawa et al. (26) found *E. coli* to be rather resistant to it. Additionally, the NO-release profiles reported were limited to a relatively short duration, lasting up to 24 h (21, 26). Moreover, the highly acidic nature (≤ pH 3) of that formulation might raise safety concerns in the case of balloon rupture, causing potential patient discomfort and tissue damage.

**Fig 1:**
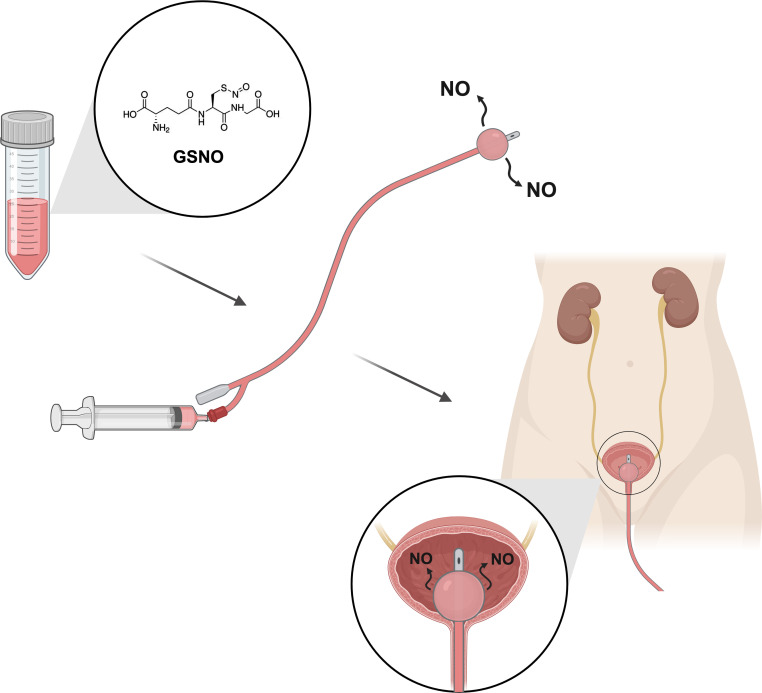
Graphical overview of the study concept. The Foley catheter balloon is inflated with an S-nitrosoglutathione (GSNO) formulation intended to provide sustained nitric oxide (NO) release for the prevention of bacterial infection.

Herein, we performed the very first proof-of-concept study evaluating the feasibility and effectiveness of a new approach utilizing the solution of the endogenous nitrosothiol (RSNO) type NO donor GSNO (S-nitrosoglutathione) as balloon inflation fluid to prevent CAUTIs via the bactericidal and bacteriostatic effects of NO (Fig. 1). GSNO is the nitrosated form of L-glutathione (GSH) tripeptide, and it is also a physiologically relevant RSNO that functions as a natural NO reservoir *in vivo* (27–29). While NO-releasing materials have been studied previously, the use of GSNO as a localized strategy to reduce CAUTI-associated bacterial colonization and biofilm formation requires further investigation. Compared with many synthetic NO donors, GSNO offers several potentially advantageous features for CAUTI prevention: (i) it is derived from glutathione, a naturally occurring intracellular antioxidant; (ii) it can release NO under physiologically relevant conditions; and (iii) it can provide localized antimicrobial activity without relying on traditional antibiotics. These properties distinguish GSNO from conventional antimicrobial catheter coatings and from other NO donor chemistries such as diazeniumdiolates, acidified nitrite solutions, or NO-releasing synthetic polymers, which have different release kinetics, stability profiles, byproducts, and biocompatibility concerns.

In this study, our aim was to demonstrate that the novel GSNO-based inflation fluid formulation for the balloon of the urinary catheters can induce sustained NO emission through the wall of the catheter balloon, while the pH of the inflation fluid is maintained at 5.5–6.5, normal for the healthy bladder (30, 31). This approach would be easily translatable, since GSNO has good storage stability in its solid form for up to 6 months as demonstrated earlier (32). Our study also evaluates for the first time whether prolonged exposure to NO from the GSNO inflation fluid causes damage to the bladder tissue in a 7-day-long porcine model.

The novelty of using GSNO to reduce CAUTI risk therefore lies in its combination of endogenous origin, NO-mediated broad-spectrum antimicrobial activity, antibiofilm potential, and suitability for localized NO delivery. Rather than acting as a conventional antibiotic, GSNO provides a non-antibiotic strategy aimed at preventing early bacterial attachment and biofilm development on catheter surfaces. This makes GSNO a promising candidate for reducing CAUTI risk and supports further investigation of its antimicrobial performance in urinary catheters and other biomedical applications.

## MATERIALS AND METHODS

### GSNO synthesis

The GSNO (MW = 336.32 g/mol) was synthesized using a previously reported protocol (33). Briefly, reduced L-glutathione (GSH, Sigma-Aldrich, St.Louise, MO) was dissolved in aqueous hydrochloric acid (Fisher Scientific, Waltham, MA) protected from light. The reaction was cooled by using ice-cold water and was continuously purged with N_2_ gas to remove any O_2_. GSH nitrosation occurred when an equal molar amount of sodium nitrite (Sigma-Aldrich, St.Louise, MO) was added. Ice-cold acetone was used to precipitate the GSNO after approximately 40 min of stirring. The pink precipitate was separated by vacuum filtration and washed by ice-cold water (3 × 10 mL) and acetone (3 × 10 mL) (Fisher Scientific, Waltham, MA), respectively. The GSNO product was then vacuum dried at room temperature for 24 h.

### Preparation of inflation fluids for Foley catheter

Inflation fluids were prepared by reconstituting 50 mM GSNO in deionized (DI) water (purity ≥18 MΩ cm at 25°C), 0.2 M 2-[4-(2-hydroxyethyl)piperazine-1-yl]ethanesulfonic acid (HEPES, free acid) (Carbiochem, Burlington, MA), and 0.2 M phosphate-buffered saline (PBS) buffer (Gibco, Paisley, Scotland). The buffer formulations also contained 100 µM ethylenediaminetetraacetic acid (EDTA) (Sigma-Aldrich, St. Louis, MO) to chelate any potential metal ion contamination that can catalyze RSNO (GSNO) decomposition (34). The pH of the inflation fluids was initially adjusted to 6.5 using sodium hydroxide (NaOH) pellets (98.5%) (Sigma-Aldrich, St. Louis, MO). Two-way, 100% silicone, 10 cubic centimeters (balloon), 14 Fr urinary Foley catheters were used for this study.

### NO flux measurements

To measure the NO emitted from the GSNO inflation fluid through the catheter balloon material, the balloon of the Foley catheter filled with the inflation fluid was immersed in 10 mM PBS (Gibco, Paisley, Scotland) with 100 µM EDTA (Sigma-Aldrich, St. Louis, MO), pH 7.4, in a custom-made glass cell (NOA cell) connected to an ozone chemiluminescence NO analyzer (NOA, GE Sievers 280i, Boulder, CO). Prior to measurement, the NOA was calibrated with zero gas (nitrogen) and 100 ppm NO balance N_2_ span gas (Airgas, Radnor, PA). The NOA cell was continuously sparged with N_2_ gas at a flow rate of 100 mL/min through a glass frit, while its headspace was maintained at atmospheric pressure and connected to an atmospheric pressure N₂ gas supply and the NOA. The sample gas flow rate was set to 200 mL/min with a mass flow controller (Alicat SS, Tucson, AZ). The NOA cell was immersed in a 37°C water bath for the duration of the measurement. The NO release from the inflated Foley catheter balloon was measured daily. The NO release was reported as NO flux units (10^−10^ mol min^−1^ cm^−2^) after normalizing the measured NO levels to the estimated surface area of the inflated catheter balloon. Between measurements, the NOA cell containing the entire Foley catheter was sealed to minimize evaporation and prevent oxygen from entering and was kept in an incubator at 37°C. At the end of the testing period, the final pH of the inflation fluid was measured (Toledo Mettler, Columbus, Ohio).

### Antibacterial study

**Fig 2:**
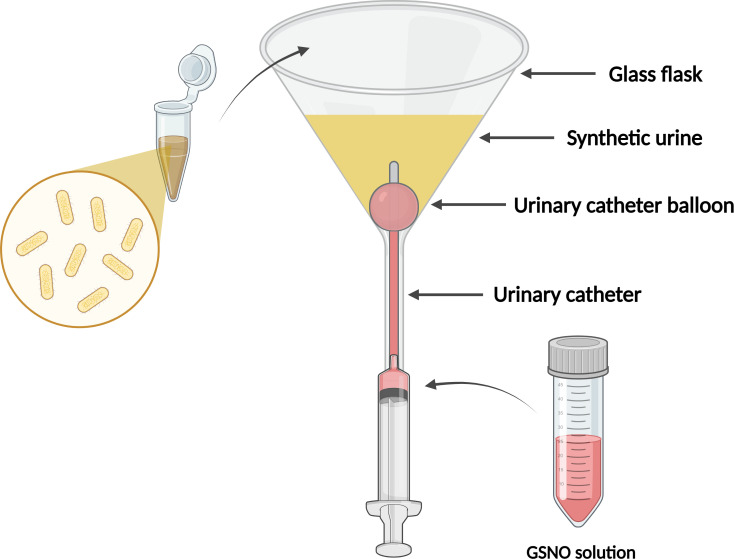
Schematic of the *in vitro* bacterial setup of the GSNO solution-inflated Foley urinary catheter in synthetic urine. The closed glass container system depicts the synthetic urine (yellow), and the urinary catheter with the inflated retention balloon is filled with the pink GSNO solution.

For the antimicrobial assay, the Foley catheter was placed into a 100 mL volumetric flask and filled with 20–25 mL of synthetic urine (8361-1, Fisher Scientific, Waltham, MA). The synthetic urine (pH 7.9) was inoculated with a uropathogenic *E. coli* (CFT073 or also known as ATCC 700928) at a final concentration of 1 × 10^6^ colony-forming unit per milliliter (CFU/mL) (Fig. 2). The bacterial strain was originally isolated from biological specimens of a female patient with pyelonephritis and is widely used as a representative uropathogenic *E. coli* strain (35, 36). The inflation fluid formulation (50 mM GSNO in 0.2 M HEPES with 100 µM EDTA) was used to inflate the NO-releasing Foley catheter balloon. HEPES buffer solution was used as a control. The Foley catheter balloon was incubated with the synthetic urine at 37°C for 7 days. After this period, bacterial colonization on the catheter balloon surface and planktonic bacterial cells in the synthetic urine were quantified using a 10-fold serial dilution and plate counting method. The antibacterial properties of the NO-releasing formulation were evaluated in comparison to the control group. One of the samples from each group (control and GSNO) was stained with a BacLight staining kit (L7012, Thermo Fisher Scientific, Ann Arbor, MI) according to the manufacturer’s instructions in the dark for 15 min to assess the degree of live (green colored) bacteria in the “biomass” on the catheter surface. Microscopic images were obtained by using a fluorescence microscope with appropriate filter sets (488/520 nm for SYTO-9 and 493/636 nm for propidium iodide).

### Preclinical porcine model

**Fig 3:**
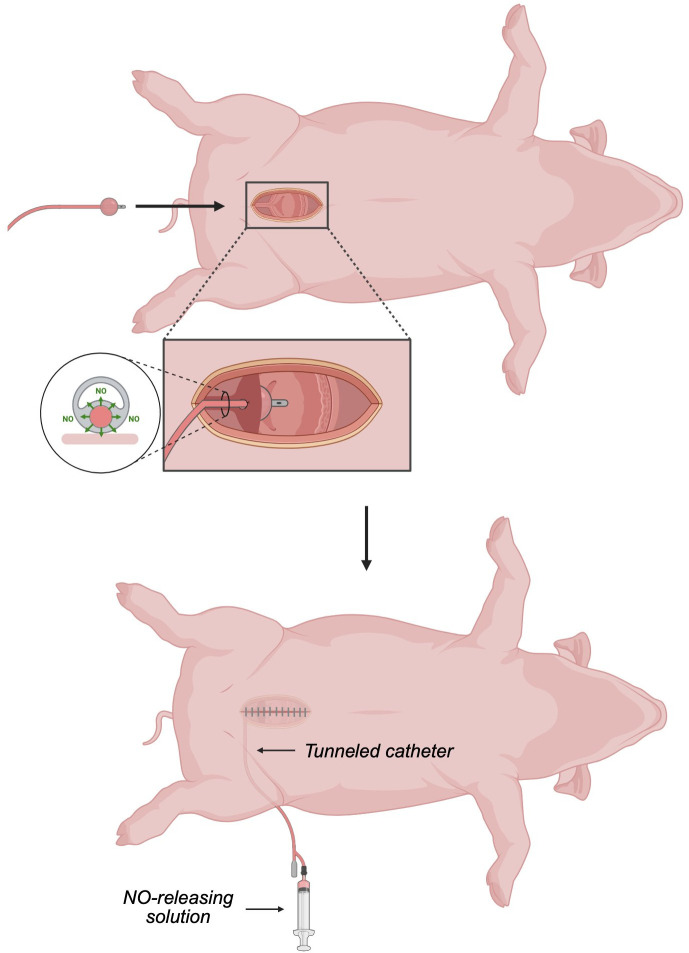
Schematic illustration of the suprapubic Foley urinary catheter implantation in a 7-day-long porcine model. NO released from the pink GSNO solution diffuses through the inflated Foley catheter material, providing an antimicrobial effect to help reduce CAUTI risk.

The potential toxicity of the NO inflation fluid on the bladder cells and surrounding tissues was evaluated using a 7-day *in vivo* porcine model (Fig. 3). Animals received humane care in accordance with the National Research Council Guide for the Care and Use of Laboratory Animals (37). This study was conducted in accordance with the University of Michigan Institutional Animal Care and Use Committee under protocol #10710.

Four (*n* = 4) juvenile swine (45–55 kg, 3–4 months of age) were used. All animals were sedated and induced for general anesthesia prior to implantation of the Foley catheter following protocols previously described by our group (38, 39). Each recovery procedure involved a minilaparotomy of the lower abdomen to surgically insert the Foley catheter into the bladder via a suprapubic approach. A 2–0 vicryl purse-string suture was placed extravesical between the adventitia and the muscularis layers of the bladder. A small cystotomy was performed to advance the sterile section of the silicone Foley catheter with the balloon (14 Fr, 10 cc) into the bladder. The Foley catheter balloon was filled with 10 cc of the novel NO-releasing GSNO liquid formulation using a sterile syringe, the purse-string suture was tied, and the catheter was secured. The remaining portion of the catheter was tunneled to the left abdomen and securely anchored to the skin to prevent displacement and maintain proper positioning throughout the study. Following the procedure, pigs were recovered from anesthesia and monitored for 24 h in the laboratory animal intensive care unit. They were then transported to a dedicated housing unit for daily care and observation during the 7-day study period. The incision site was regularly examined for signs of edema, tenderness, bleeding, or infection. The animals’ overall well-being was assessed using a pain scale from 1 to 10 by monitoring indicators such as lethargy, depression, anorexia, and vocalization. Transdermal fentanyl patches (1–10 µg/kg) were placed 12–24 h prior to skin incision; if any additional pain control was needed, buprenorphine (0.005–0.02 mg/kg) was administered. After the 7-day period, the animals were sedated using general anesthetics and euthanized with sodium pentobarbital. The NO liquid formulation was drained from the Foley catheter balloon to measure the pH of the drained GSNO solution. The balloon was then reinflated with 10 mL of normal saline to ensure the catheter remained in position for further tissue analysis. The entire bladder, with the Foley catheter *in situ*, was harvested and placed into 10% neutral-buffered formalin (Fisher Scientific, Waltham, MA) for preservation.

### Histopathological interpretation method

The proof-of-concept porcine model was used to assess any histopathological effect of the NO-releasing Foley catheter. Histological assessment of the bladder was performed by the Pathology Core at the University of Michigan Unit for Laboratory Animal Medicine (RRID:SCR 018823). After fixation, tissues were trimmed to represent all areas of the bladder, including the catheter entry point. Trimmed tissues were cassetted and processed to paraffin in an automated tissue processor (TissueTek VIP5, Sakura Finetek, Torrance, CA). Processed tissues were embedded in paraffin and sectioned at 4 µm on a rotary microtome (Leica Biosystems, Buffalo Grove, IL). Tissues were mounted on glass slides and stained with hematoxylin and eosin using routine protocols on an automated histostainer (Leica ST5010 Autostainer, Leica Biosystems), followed by coverslipping. Tissues were evaluated by light microscopy, and interpretation of tissue damage was performed by board-certified veterinary pathologists. Representative images were taken from whole slide images generated on a digital slide scanner (Leica Aperio AT2, Leica Biosystems). Composite image construction was performed in Adobe Photoshop (v26.8.1, Adobe Inc., San Jose, CA). Image adjustments were confined to global adjustments of brightness, contrast, and white balance. Photo edits were confined to cropping of background elements that did not affect image interpretation.

### Statistical analysis

Statistical evaluation of the experimental data was performed using GraphPad Prism v10.4.1 (GraphPad Software, Boston, MA). Comparisons between two groups were analyzed using Student’s *t*-test. Comparisons involving more than two groups were analyzed using one-way ANOVA followed by Tukey’s post-hoc analysis. A *P* value < 0.05 was deemed statistically significant. Text and figure legends specify the number of replicates (*n* ≥ 3) and definitions corresponding to each data set. Data are presented as mean ± standard deviation (SD).

## RESULTS

### NO generation from the inflated Foley catheter

**Fig 4:**
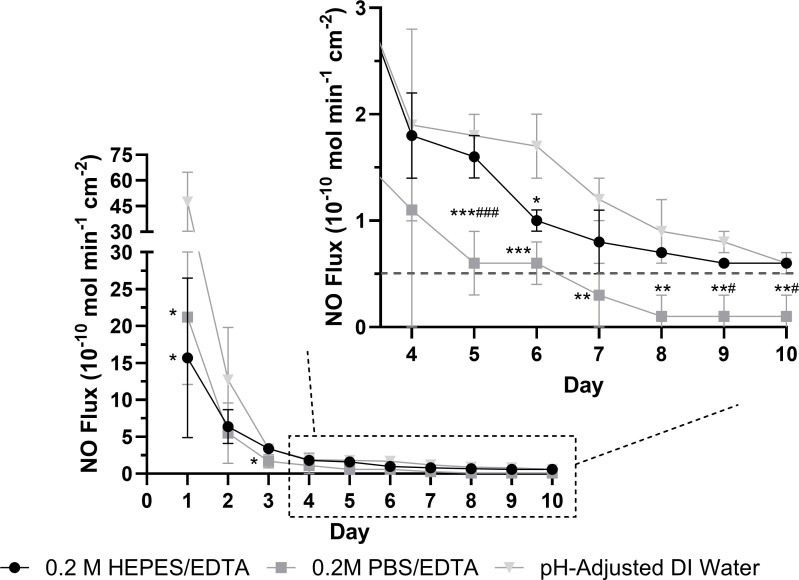
NO release profile of the GSNO formulations. NO-release profile of 50 mM GSNO in different dissolving agents (DI water, PBS, and HEPES buffers) over a 10-day period (initial pH 6.5). Data represent mean ± (SD) and *P < 0.05, **P < 0.01, ***P < 0.001 (vs DI water)*, ^#^P <* 0.05*, ^##^P* < 0.01*, ^###^P* < 0.001 (vs HEPES*)*.

The primary goal was to develop a simple NO-releasing formula that could be easily reconstituted with the intention of streamlining its possible integration into the healthcare system. First, the NO-release profile and the pH change of the GSNO solution in DI water (pH adjusted to 6.5 with NaOH) were measured (Fig. 4). The pH of the solution decreased from 6.5 to 5.1 ± 0.2, below the minimum target level of pH 5.5. To better control the pH, PBS and HEPES buffers were tested with 50 mM GSNO. The buffered solutions provided NO release of >0.5 × 10^−10^ mol min^−1^ cm^−2^ for 6 and 10 days, respectively (Fig. 4). The NO-release profile of the GSNO/PBS was significantly different when compared to GSNO/DI water on day 1 (*P* < 0.05, *n* ≥ 3), day 3 (*P* < 0.05, *n* ≥ 3), day 5 (*P* < 0.001, *n* ≥ 3), day 6 (*P* < 0.001, *n* ≥ 3), days 7–9 (*P* < 0.001, *n* ≥ 3), and day 10 (*P* < 0.05, *n* ≥ 3) (Fig. 4). The GSNO/HEPES had an overall very similar NO-release profile compared to GSNO/DI water except on days 1 (*P* < 0.05, *n* ≥ 3) and 6 (*P* < 0.001, *n* ≥ 3). Both buffered GSNO formulations stayed within the desired pH range of 5.5–6.5 at the end of the NO-release measurement (PBS: 6.3 ± 0.2; HEPES: 6.0 ± 0.1). Hence, for the rest of the study, we used 50 mM GSNO solution in 0.2 M HEPES buffer with an initial pH value of 6.5.

### Antibacterial potential of the NO-releasing inflation fluid

**Fig 5:**
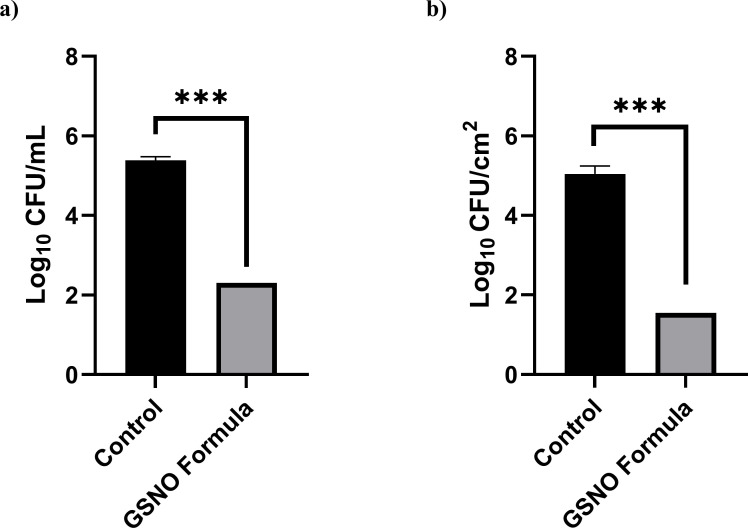
Antibacterial performance of the NO-releasing liquid formulation. (**a**) Viable cell count of free-floating bacteria (CFU/mL) between Control and GSNO (50 mM in 0.2 M HEPES) catheter solutions (***P < 0.001). (**b**) Viable cell count adhered to the surface of the catheter balloon (CFU/cm^2^) between Control and GSNO (50 mM in 0.2 M HEPES) catheter solutions (***P < 0.001). Values are shown as mean ± SD (*n* = 3).

**Fig 6:**
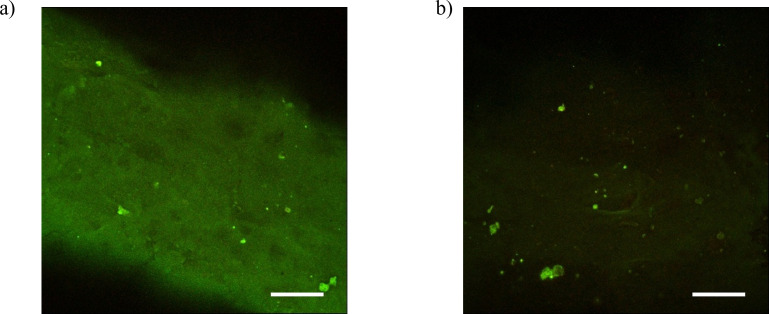
Fluorescent imaging of antimicrobial assays. Representative fluorescent images of bacterial colonization on the (**a**) Control and the (**b**) GSNO/HEPES Foley catheter balloon (Scale bar: 20 µm).

During the *in vitro* antibacterial test, the optimal GSNO formulation demonstrated excellent antibacterial properties when tested against *E. coli* for 7 days. The GSNO formulation demonstrated a statistically significant (*P* < 0.001) 3-logarithmic reduction (2.0 × 10^2^ ± 3.1 × 10^1^ CFU/mL, *n* = 3) in free-floating, planktonic bacteria count when compared to Control formulation (2.5 × 10^5^ ± 5.0 × 10^4^ CFU/mL, *n* = 3) (Fig. 5a). Furthermore, looking at the count of the bacteria attached to the balloon surface, the GSNO formulation showed a significant (*P* < 0.001) 4-logarithmic reduction (3.6 × 10^1^ ± 5.4 × 10^2^, *n* = 3) (Fig. 5b) compared to Control (1.2 × 10^5^ ± 5.4 × 10^4^ CFU/cm^2^, *n* = 3) formulation. The bacteria colonization on the catheter surfaces is depicted by the representative microscopy images of Fig. 6a (Control) and Fig. 6b (GSNO/HEPES).

### *In vivo* safety study of the NO-releasing inflation fluid

**Fig 7:**
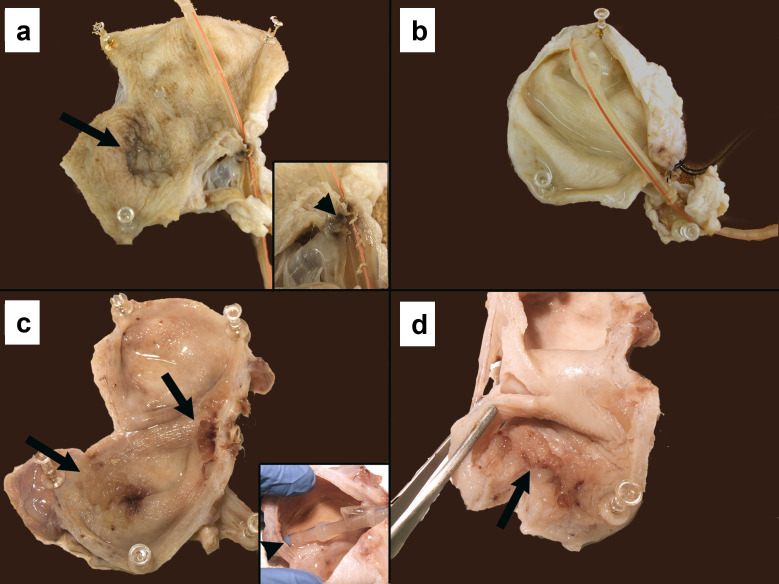
Representative histological images from the bladder body for pigs 1–4 (**a–d**). Images show opened bladders with (**a and b**) or without (**c and d**) catheters in place. Arrows denote sites where the catheter is in direct contact with the bladder wall, corresponding to localized mucosal thickening (a) or focal polypoid mucosal hyperplasia (**c and d**). Inset ‘a’ also shows localized hemorrhage (arrowhead) at the point of catheter entry in the animal that had experienced catheter rupture. Inset ‘c’ shows a contact point (arrowhead) of the distal catheter tip on the bladder wall prior to fully opening the bladder. This contact point corresponded to an area of hyperplasia (arrows).

**Fig 8:**
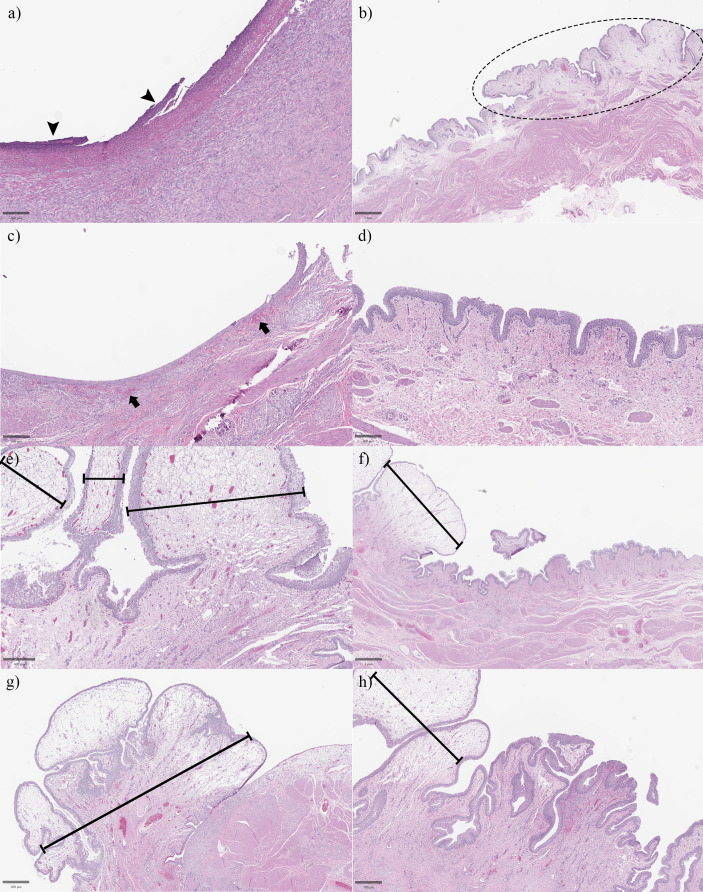
Representative histopathologic images of bladders from pig 1 (**a and b**), pig 2 (**c and d**), pig 3 (**e and f**), and pig 4 (**g and h**), demonstrating focal, mild mechanical damage limited to sites of direct catheter contact. Left panels (**a, c, e, and g**) show mucosa at catheter contact points (points of entry or catheter tip), right panels (**b, d, f, and h**) represent the majority of the bladder body mucosa. (**a**) Catheter contact point in pig 1 with mucosal necrosis (arrowheads) and inflammation. (**b**) Mild mucosal hyperplasia (circle) and normal mucosa in the remainder of the bladder in pig 1. (**c**) Catheter contact point in pig 2 with focal mild hemorrhage and inflammation (arrows). (**d**) Normal mucosa in the body of the bladder for pig 2. (**e**) Catheter contact point with focal hyperplasia and edema (brackets) in pig 3. (**f**) Mild focal hyperplasia and normal mucosa in the bladder body in pig 3. (**g**) Catheter contact point in pig 4 showing focal mucosal hyperplasia (bracket), with edema in pig 4. (**h**) Mild, focal mucosal hyperplasia (bracket) and normal mucosa in the bladder body for pig 4. Hematoxylin and eosin staining. Bars 200 µm (**a, c, and d**); 400 µm (**e**); 500 µm (**g and h**); and 1 mm (**b and f**).

At the end of the study (day 7), necropsy revealed that one (*n* = 1) Foley catheter balloon had ruptured during the experiment, although the exact time of failure was unknown. All other (*n* = 3) Foley catheters and GSNO-based solutions (pH 5.6 ± 0.1 post-study) were recovered without any damage. Gross findings of all four studies (Fig. 7a through d), including the balloon rupture (Fig. 7a), were confined to mucosal thickening and focal hemorrhage at the site of the Foley catheter entry or at the mucosal point of contact of the catheter tip (Fig. 7, arrows). The remainder of the bladder mucosa was grossly normal. Likewise, histological findings (Fig. 8a through h) were confined to the catheter entry point and points of mucosal contact. Findings at mucosal contact points (Fig. 8a, c, e and g) included localized mucosal hyperplasia and edema as well as localized foci of inflammation, necrosis, and hemorrhage. In contrast to these localized changes, bladder mucosa at sites away from the entry point or the distal point of contact was histologically normal (Fig. 8b, d, f and h). The lack of mucosal findings away from the sites of catheter entry or contact was consistent with a lack of toxicity attributable to NO elution itself.

## DISCUSSION

The data presented herein demonstrate that a Foley catheter balloon inflated with a GSNO-based liquid formulation can generate an adequate NO flux through the wall of the catheter balloon for an extended period up to 10 days. This new, simple and translatable approach has the potential to prevent fatalities and greatly reduce the costs associated with CAUTIs.

It is known that low levels of NO are already produced by cells that line the urinary tract (40) via the enzyme NO synthase (NOS), which creates NO from L-arginine (41). Our laboratories have developed novel NO-emitting materials by solvent impregnation of the S-nitrosothiol type NO donors into the polymers to mimic the NO release of the normal endothelium (42). This technology was successfully used to prevent biofilm and thrombus formation in extracorporeal circuits without systemic side effects. In addition, previous studies with NO-emitting intravascular catheters have also demonstrated significant antimicrobial effects (43–45). Colletta et al. (23) have also successfully used the solvent impregnation method in modifying an entire Foley catheter to prevent biofilm formation (23). Although the technology showed great promise, the mechanical properties of the impregnated catheter were not evaluated, raising concerns that the known crystal formation (24, 25) from the NO donor, SNAP, could compromise the catheter’s mechanical properties and hinder the product’s overall clinical translatability. Our proposed GSNO-based, NO-releasing inflation fluid formulation does not modify or compromise the material of the Foley catheter, but it relies on the excellent permeability of the catheter balloon material for NO.

Our primary objective was to develop and test the feasibility and safety of an inflation fluid formulation for the Foley catheter capable of sustaining NO release for a minimum of 7 days while maintaining the pH within the optimal range for a healthy urothelial environment (pH 5.5–6.5). The target NO-release threshold for a minimum of 7 days was selected to prevent bacterial colonization early on. According to previous literature (46) and data summarized in the Centers for Disease Control and Prevention (CDC) report (47), the risk of indwelling Foley catheter-related bacteriuria increases rapidly within the first couple of days after catheter placement. Therefore, maintaining sufficient antimicrobial activity through sustained NO release is particularly critical during this early period and may provide continued protection beyond it.

It is well-known that GSNO’s rate of NO release is pH-dependent (48, 49). At lower pH, GSNO solutions tend to release NO more rapidly due to the acid-catalyzed decomposition of GSNO. Conversely, at higher pH, the rate of NO release is slower (48, 49). Additionally, the acidic pH of the unbuffered aqueous solution of GSNO (pH ~3.4) raised concerns about the potential risk associated with an accidental balloon rupture, when the inflation fluid comes into direct contact with the surrounding tissue and potentially causes damage to it. This was also concerning in earlier studies (21, 26) utilizing an acidified NO donor (DETA NONOate) and nitrite solutions, where the NO release only lasted for 24 h, and the pH fell below 3. To address these concerns in our study, we conducted tests on pH-adjusted DI water and buffer solutions with more physiological pH levels.

The pH-adjusted (initial pH 6.5) solution of 50 mM GSNO in DI water and HEPES buffer exhibited a sustained NO release for at least 10 days, while in PBS buffer, the NO flux decreased below the target level within 6 days. This difference is most likely attributable to variations in the buffer capacity of the different solutions. Additionally, the NO release duration may be further shortened *in vivo* by the reaction of NO with the phosphate in the presence of O_2_ (50). Xu et al. (2013) reported that N₂O₃ readily reacts with H₂PO₄⁻ or HPO₄²⁻ through a five-membered cyclic transition state (50).

The GSNO solution in DI water had no buffer capacity at the initial pH of 6.5; thus, unsurprisingly, it failed to maintain the target pH range. In contrast, in PBS, it maintained the pH within the target range throughout the study due to its higher buffer capacity. Also, the HEPES buffer had adequate buffer capacity (pH 6.1 ± 0.04 post-study) and provided an overall more sustained and desirable NO-release profile *in vitro* compared to the DI water or the PBS formulations. Although HEPES has suboptimal buffer capacity at pH below 6.5, at the relatively high concentration (0.2 M), it was adequate to maintain the pH above 5 during the test period of up to 10 days. We speculate that the lower buffer capacity of the HEPES solution might contribute to the more sustained NO release compared to the PBS buffer solution. Consequently, we chose the HEPES formulation for the antibacterial and *in vivo* study.

CAUTIs are caused by a number of different pathogens including gram-negative bacteria, gram-positive bacteria, and fungi. Common CAUTI-related bacteria are *Enterococcus spp*. (11%), *Klebsiella pneumoniae* (8%), *Candida* spp. (7%), and *Staphylococcus aureus* (3%) (51). However, the most prevalent pathogen is uropathogenic *E. coli*, making up nearly 70% of the infections (52). One of the key factors in the development of CAUTI is the duration of catheterization. Prolonged catheter placement significantly increases the risk of bacterial colonization and subsequent infection (53). Thus, demonstrating the GSNO formulation’s sustained NO release and its 3- and 4-logarithmic reductions in free-floating planktonic and retention balloon surface-adhered *E. coli* cell counts, respectively, after a 7-day period was critical. These results highlight the GSNO formulation’s sustained bactericidal properties.

This study also demonstrated that the NO released from the GSNO-based formulation did not cause tissue toxicity, as evidenced by the absence of diffuse gross or microscopic bladder mucosal damage in a preclinical porcine model. Even in the single instance of accidental rupture of the Foley retention balloon, microscopic examination revealed no damage to the surrounding tissues. Additionally, the pathological findings at catheter entry and contact sites were consistent with expected localized mechanical irritation of the tissue due to the surgical procedure. Similar mucosal hyperplasia and localized inflammation at mucosal contact points have been previously reported with standard (non-NO-releasing) catheters, even within days of catheterization (54–56). These contact-associated mucosal findings are typically reversible (54). Thus, the histological findings showed no evidence of diffuse mucosal damage (as would be expected with toxicity) and only localized changes at catheter contact or entry points.

The pH of the GSNO solution in HEPES decreased to pH 5.6 ± 0.1 during the *in vivo* study, which is lower than the observed pH *in vitro* (6.0 ± 0.1). A potential explanation is the temperature variation: the *in vitro* experiments were conducted at 37°C, while the porcine model typically involves a naturally higher temperature, approximately 39°C (57). The higher temperature can accelerate the GSNO decomposition, causing the increased pH to drop *in vivo*. Additionally, we speculate that the O₂ present *in vivo* could also have an effect compared to the *in vitro* NO release studies conducted in more anaerobic conditions. During *in vitro* measurements, the solution is constantly sparged with N₂, which also removed any O_2_ from the catheter balloon through its silicone wall that is highly permeable to O₂. In contrast, the *in vivo* conditions involve aerobic conditions, where the O₂ can react with NO, forming NO₂, N_2_O_3_, and eventually nitrous acid (HNO_2_), which further decreases the pH.

Despite the promising findings, the study has certain limitations. Future research should include comprehensive *in vitro* testing of the GSNO formulation, particularly focusing on refining buffer and NO donor concentrations, as well as pH, to achieve more controlled NO release. These optimizations should minimize the osmotic pressure of the inflation fluid and reduce the likelihood of crystal formation from the buffer components. These studies will also evaluate the antimicrobial performance of the described technology against a broader panel of CAUTI-associated uropathogens. Storage stability of the optimized formulation should also be evaluated in the future. Storage requirements, however, may differ depending on clinical needs: for applications where long-term storage is necessary, a two-component kit could be utilized, separating the dry (e.g., GSNO) and liquid (buffer) components. Conversely, for situations requiring frequent or immediate use (33, 58), the reconstituted solution may be stored short-term at lower temperatures (at or below room temperature).

Additional limitations include the overall small sample size of four animals, the exclusive use of male pigs, and the lack of an independent control group, which may limit the generalizability of the current findings. Furthermore, the Foley catheter was placed surgically via a suprapubic approach, which was necessary to ensure the catheter patency and prevent its premature removal by the animal, although it differs from the typical clinical placement. Future studies will address these limitations by including appropriate control and experimental groups, increasing the sample size to a minimum of *n* = 3 per group, and ensuring balanced representation of animal sex.

Although sustained NO release from the GSNO-based inflation fluid showed antimicrobial activity, urinary protein deposition on the catheter surface was not directly assessed. Because fibrinogen and other host-derived proteins can promote bacterial adhesion and biofilm formation (59), future studies should evaluate whether NO release alters fibrinogen deposition and reduces bacterial colonization.

Overall, this study introduces a promising and translatable approach to addressing a significant and prevalent healthcare issue, CAUTI. This strategy has the potential to reduce the risk of infection, a common complication associated with urinary catheterization due to its sustained bactericidal and bacteriostatic effect. Together, these benefits may contribute to the development of NO-based technologies aiming to improve patient outcomes and reduce overall healthcare costs.

### Conclusion

In conclusion, this manuscript demonstrates that a GSNO-based, NO-releasing solution can be safely and effectively used to inflate urinary Foley catheter balloons for extended periods without inducing toxicity. This biocompatible and user-friendly intervention enables extended NO release through the catheter balloon wall, showing promise in preventing CAUTI. The sustained NO release not only inhibits bacterial colonization but also aligns with safety and usability requirements in clinical practice. By leveraging this simple yet innovative technology, healthcare systems could significantly reduce the economic burden of CAUTIs and prevent many of the associated fatalities each year.

## Data Availability

The data presented in this study will be made publicly available as part of the published article and may be obtained from the corresponding author upon reasonable request.
